# MPXV DNA kinetics in bloodstream and other body fluids samples

**DOI:** 10.1038/s41598-024-63044-5

**Published:** 2024-06-12

**Authors:** Silvia Meschi, Francesca Colavita, Fabrizio Carletti, Valentina Mazzotta, Giulia Matusali, Eliana Specchiarello, Tommaso Ascoli Bartoli, Annalisa Mondi, Claudia Minosse, Maria Letizia Giancola, Carmela Pinnetti, Maria Beatrice Valli, Daniele Lapa, Klizia Mizzoni, David J. Sullivan, Jiangda Ou, Daniele Focosi, Enrico Girardi, Emanuele Nicastri, Andrea Antinori, Fabrizio Maggi

**Affiliations:** 1 Laboratory of Virology, National Institute for Infectious Diseases, Lazzaro Spallanzani IRCCS, 00149 Rome, Italy; 2 Clinical and Research Infectious Diseases Department, National Institute for Infectious Diseases, Lazzaro Spallanzani IRCCS, 00149 Rome, Italy; 3 Scientific Direction, National Institute for Infectious Diseases, Lazzaro Spallanzani IRCCS, 00149 Rome, Italy; 4 Department of Molecular Microbiology and Immunology, Johns Hopkins Bloomberg School of Public Health, Baltimore, MD 21205 USA; 5 Brain Injury Outcomes, Department of Neurology, Johns Hopkins University School of Medicine, Baltimore, MD 21202 USA; 6 North-Western Tuscany Blood Bank, Pisa University Hospital, Pisa, Italy

**Keywords:** Virology, Infectious diseases

## Abstract

Since spring 2022, the global epidemiology of the monkeypox virus (MPXV) has changed. The unprecedented increase of human clade II MPXV cases worldwide heightened concerns about this emerging zoonotic disease. We analysed the positivity rates, viral loads, infectiousness, and persistence of MPXV DNA for up to 4 months in several biological samples from 89 MPXV-confirmed cases. Our data showed that viral loads and positivity rates were higher during the first two weeks of symptoms for all sample types. Amongst no-skin-samples, respiratory specimens showed higher MPXV DNA levels and median time until viral clearance, suggesting their usefulness in supporting MPXV diagnosis, investigating asymptomatic patients, and monitoring viral shedding. Infectious virus was cultured from respiratory samples, semen, and stools, with high viral loads and collected within the first 10 days. Notably, only one saliva and one semen were found positive for viral DNA after 71 and 31 days from symptoms, respectively. The focus on bloodstream samples showed the best testing sensitivity in plasma, reporting the overall highest MPXV DNA detection rate and viral loads during the 3-week follow-up as compared to serum and whole-blood. The data here presented can be useful for MPXV diagnostics and a better understanding of the potential alternative routes of its onward transmission.

## Introduction

From January 2022 to October 17, 2023, more than 91,190 laboratory-confirmed cases of clade II monkeypox virus (MPXV), including 157 deaths, have been reported to the World Health Organization (WHO) from 115 countries in all six WHO Regions^1^. Despite a declining incidence, MPXV is still diagnosed worldwide. The highest cumulative number of cases, during the 2022–2023 outbreak, has been reported in the United States of America, Canada, Brazil, Colombia, Mexico, and Peru in the Americas; Spain, England, and Germany in the European Region; and in China, Japan, and the Republic of Korea in the Western Pacific Region^1^. Concern is emerging about the clade I MPXV, known for its higher severity and mortality, as increased spreading in the Democratic Republic of Congo has been recently reported and, for the first time, sexual contact was documented among the routes of transmission^2^. The MPXV usually spreads through close skin-to-skin contact, including direct contact with cutaneous lesions, scabs, or body fluids from a person with MPXV, but it can also be transmitted via respiratory secretions, contact with contaminated fomites and surfaces, or infected animals^3^. During the recent clade II MPXV outbreak, of all reported modes of transmission, skin and mucosal contact during sexual intercourse was the most prevalent. Understanding the viral dynamics of the early stage of infection and the persistence of MPXV DNA in body fluids contributes to examining its dissemination and human-to-human transmission. Recent studies reported data on the detection of MPXV DNA in various samples^4–9^. However, the information on how frequently, at which viral load, how infectious, and for how long, MPXV can be detected in the different clinical samples is still limited and varies amongst the studies^4,6,10,11^. Data on viral shedding and its kinetics are essential to inform public health strategies and improve clinical diagnostic testing, for the purpose of identifying MPXV patients and limiting transmission events. Since conditions other than MPXV can cause similar skin rashes and MPXV itself may determine atypical manifestations, the identification of MPXV disease on the basis of the clinical presentation alone can be difficult. The confirmation of MPXV infection relies on the detection of viral DNA by PCR in skin lesion material; however, in the absence of skin lesions, other samples can be useful^12,13^. MPXV infection is characterized by a primary viremia following the viral entry, which depicts the 1–2 week incubation period and leads to viral dissemination to the local lymph nodes, and by a secondary viremia lasting 1–3 days during the prodromal stage, when the viral load reaches the distant lymph nodes and other organs through circulation. Infected patients may be infectious during the secondary viremia phase^14^. Although these two viremic phases^14^, samples from the bloodstream are not commonly used for the diagnostic purposes of MPXV. In fact, these samples have been shown to contain low levels of viral DNA, and the frequency of MPXV DNA detection in blood differs in the studies published during the 2022 outbreak, ranging from 7% as reported by Thornhill et al. to 80% (plasma) by Coppens et al.^13,15^. The timing of sample collection and the assays carried out for DNA detection, which can have a variable low limit of detection (LLOD), differ amongst the studies; however, whether the results might be influenced by the choice of the blood matrices (whole blood vs. plasma vs. serum) tested has never been assessed for MPXV.

The aim of the present study was to extend the analysis previously performed^9^ on the frequency, viral loads, infectiousness, and persistence of clade II MPXV DNA in several biological samples to a larger cohort of MPX-patients diagnosed during 2022. In this cohort, we also investigated the clade II MPXV DNA detection in the three matrices of the blood (whole blood, serum, and plasma) to evaluate the most informative clinical samples.

## Results

### Detection and persistence of MPXV DNA in body fluids

**Figure 1:**
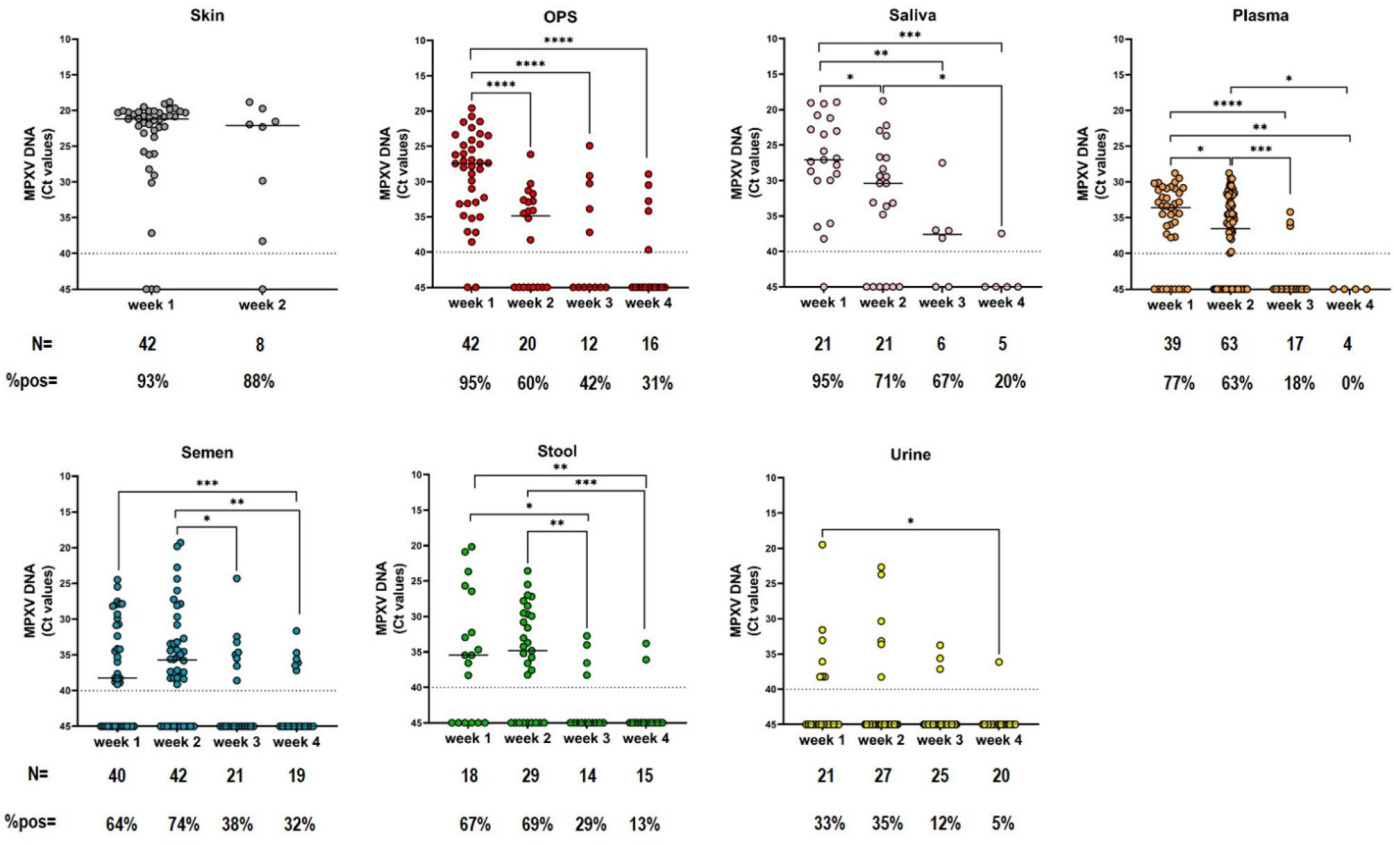
MPXV DNA levels and percentage of positive samples detected at different weeks from the onset of symptoms in each body fluid sample. Median MPXV DNA Ct values are shown for each sample type at different weeks from the onset of symptoms. The horizontal dashed line represents the limit of detection (Ct: 40); values ≥ 40 are considered negative. Statistical analysis by Mann–Whitney test between each time-point pair: *p < 0.05; **p < 0.005; ***p < 0.001; ****p < 0.0001.

During the first week, MPXV DNA was detected in different body fluids, with the highest median levels of viral DNA found in skin lesions (Ct = 21.2, 95% CI 20.5–22.3), followed by oropharyngeal swab (OPS) (Ct = 27.4, 95% CI 26.1–31) and saliva (Ct = 27.1, 95% CI 22.8–30). Median Ct values above 30 were detected in plasma (Ct = 33.4, 95% CI 31.1–35.9), stool (Ct = 35.5, 95% CI 26.4–> 40), and semen (Ct = 38.3, 95% CI 34.2–> 40) samples, while urine samples were those with the lowest MPXV DNA levels (median Ct =  > 40, 95% CI 38.2–> 40) (Fig. 1).

**Figure 2:**
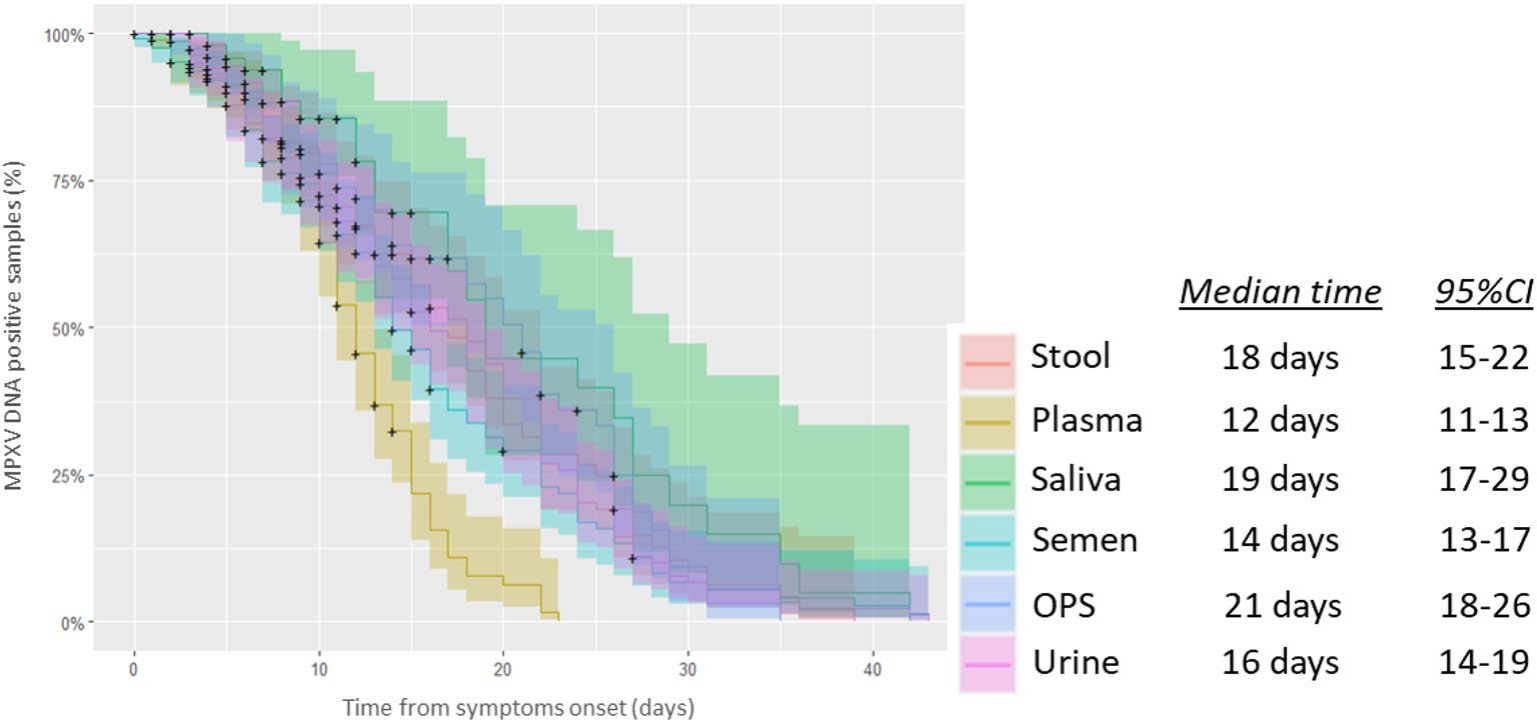
Time from symptom onset to MPXV clearance in different body fluids by the Weibull regression model. The empirical cumulative distribution function with an upper and lower 95% confidence interval and Weibull regression are shown in the graph (left). The median number of days to MPXV DNA undetectability and the 95% CI are reported near the related specimen type (right).

As shown in Fig. 1, skin samples, saliva, and OPS showed the highest positivity rates (> 90%) during week 1. Monitoring the shedding over time up to week 4 from symptoms, viral loads, and positivity rates decreased markedly in all sample types (Fig. 1). During week 4, MPXV DNA was still detected in OPS (31%), semen (32%), saliva (25%), and stool (13%), but undetected in all plasma samples tested; only one urine sample resulted positive during week 4 (5%; at 28 days FSO, Ct = 36.1). A lower proportion of positive samples was found in urine at each time point (Fig. 1). Of note, all 121 samples collected after 30 days up to 143 days FSO resulted negative for MPXV DNA, except for saliva and semen, for which we detected viral DNA in one semen sample at 31 days FSO (Ct = 35.9) and in one saliva sample at 71 days FSO (Ct = 39.2) collected from two different patients. As shown in Fig. 2, we calculated the median time from symptom onset to viral clearance for each sample type. We observed undetectability of MPXV DNA after a median of 21 (95% CI 18–26) days in OPS, followed by saliva and stool with 19 (95% CI 17–29) and 18 (95% CI 15–22) days, respectively. In semen and urine, the median time FSO to viral clearance was 14 (95% CI 13–17) and 16 (95% CI 14–19) days, respectively, while plasma samples showed a shorter median time till viral clearance with 12 (95% CI 11–13) days FSO.

#### Infectiousness of MPXV in different body fluids

The presence of infectious virus in non-skin samples was determined by attempting the recovery of replication-competent virus in cell culture by isolation from a total of 45 samples, including 13 plasma (median Ct value: 30.9, IQR 30–31.1; median DSO: 10, IQR 6–11), 11 sera (median Ct value: 31.6, IQR 30.5–32; median DSO: 8, IQR 5–5.5), 11 semen (median Ct value: 27.9, IQR 25.2–29.5; median DSO: 10, IQR 7.5–11.5), 4 OPS (median Ct value: 23, IQR 21.5–25.2; median DSO: 11.5, IQR 9–12), 3 stool (median Ct value: 22.1, IQR 21–24.2; median DSO: 11, IQR 8–12), and 3 saliva (median Ct value: 23.9, IQR 22.1–28.3; median DSO: 14, IQR 13.5–16.3), collected from 10 patients at different days. As shown in Table 1, a replication-competent virus was successfully isolated from 6 samples collected from different patients, including 1 saliva sample, 2 OPS samples, 2 semen samples, and 1 stool sample. The samples resulted positive for the presence of replication-competent virus were collected after a median time of 11 days from symptoms (IQR 6–11), and had a median MPXV DNA Ct value of 22.4 (IQR 21–23). Of the 13 plasma and 11 serum samples tested, none resulted in a positive viral culture.

**Table 1:** Viral isolation in cell culture from non-skin samples.

Sample type	N°	Ct range days	FSO range	N° of positive isolate (%)	Ct of positive isolate	FSO of positive isolate
Saliva	3	20.2–29.7	13–17	1 (33)	20.2	13
OPS	4	17.6–31.4	3–16	2 (50)	17.6/23.1	3/12
Semen	11	22.7–30.8	4–16	2 (18)	22.7/29.3	4/12
Stool	3	19.8–22.1	5–13	1 (33)	22.1	11
Plasma	13	28.7–33.2	2–14	0	n.a	n.a
Serum	11	30.2–32.7	2–12	0	n.a	n.a

N°, total number of samples tested on viral culture; FSO, days from symptom onset; n.a., not available.

#### Comparison of MPXV DNA detection in the three matrices of blood: serum versus plasma and whole blood

**Figure 3:**
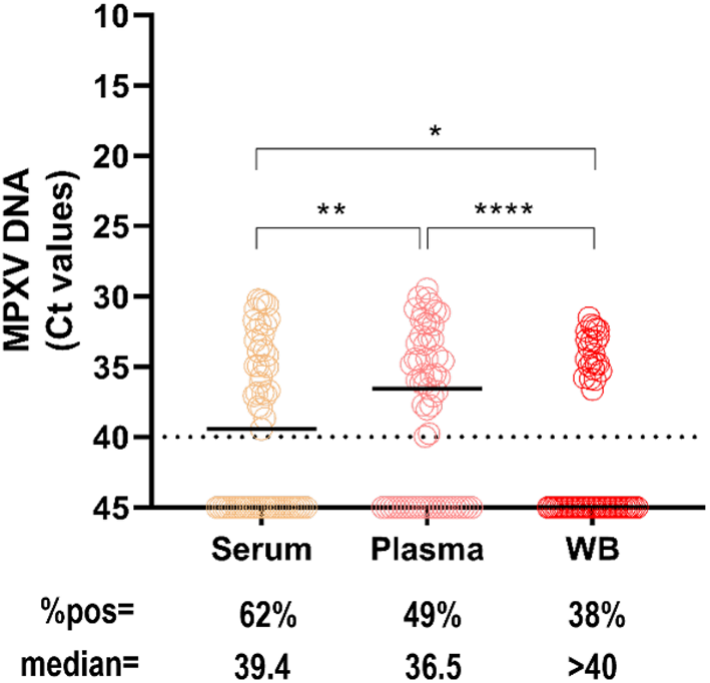
Viral loads and percentages of positivity in paired samples of serum vs. plasma vs. WB from 13 MPXV patients. Median MPXV DNA Ct values are shown for serum (n = 61), plasma (n = 61), and WB (n = 61). The horizontal dashed line represents the limit of detection (Ct = 40); values ≥ 40 are considered negative. Statistical analysis by Wilcoxon matched-pairs signed rank test: *p < 0.05; **p < 0.005; ****p < 0.0001.

We compared the three matrices of bloodstream samples, by testing paired plasma, WB and sera. Over 61 blood-paired samples collected during the 3 weeks after the symptom’s onset, we detected MPXV DNA in 38 (62.3%) plasma samples, 30 (49.2%) serum samples, and 23 (37.7%) WB (Fig. 3). Plasma samples showed the highest viral loads (plasma vs. serum, *p* = 0.0091; plasma vs. WB, *p* < 0.0001), with a median Ct value of 36.5 (95% CI 34.8–> 40) as compared to serum showing a median Ct of 39.4 (95% CI 36.7–> 40) and WB, for which the majority of the corresponding samples resulted in negative results (median Ct = 40, 95% CI 36.7–> 40).

As shown in Table 2, we obtained discordant results amongst the different blood matrices for 26 paired samples with a median Ct value of 36.1 (95% CI 35.5–36.9) in the positive samples. Among these, MPXV DNA was mostly found in plasma, with 11 samples positive in plasma only but negative in the corresponding WB and serum; five samples resulted positive in serum only and 6 in the serum-plasma pairs.

**Table 2:** Comparison of MPXV-PCR results (Ct value) in paired samples of serum, plasma, and WB.

Sample ID	Serum	Plasma	WB	FSO range
1	37.8	und	und	Week 1
2	und	37.7	und	Week 1
3	und	und	36.6	Week 1
4	36.8	und	und	Week 2
5	39.4	34.4	und	Week 2
6	37.0	37.8	und	Week 2
7	34.9	35.6	und	Week 2
8	38.7	und	und	Week 2
9	und	35.5	und	Week 2
10	und	35.7	und	Week 2
11	und	34.8	und	Week 2
12	und	39.9	und	Week 2
13	37.9	34.5	und	Week 2
14	und	33.3	und	Week 2
15	und	36.5	35.8	Week 2
16	und	38.0	und	Week 2
17	und	39.8	und	Week 2
18	und	37.1	und	Week 2
19	und	35.9	34.1	Week 2
20	34.9	34.4	und	Week 2
21	36.1	und	und	Week 2
22	33.9	36.1	und	Week 3
23	und	36.7	und	Week 3
24	34.9	und	und	Week 3
25	und	36.2	und	Week 3
26	36.9	und	34.5	Week 3

WB, whole-blood; FSO, days from symptoms onset; und, undetected.

**Figure 4:**
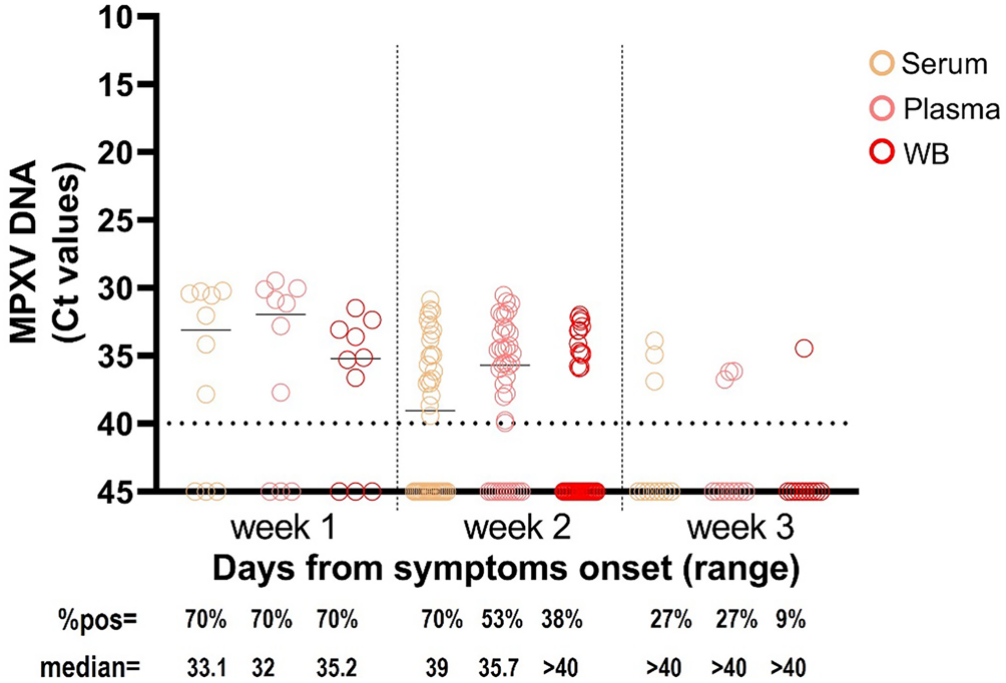
Viral loads and percentages of positivity in paired samples of serum vs. plasma vs. WB collected at different time-ranges from symptoms onset. Median MPXV DNA Ct values are shown for serum, plasma, and WB at week 1 (n = 10), week 2 (n = 40), and week 3 (n = 11). The horizontal dashed line represents the limit of detection (Ct = 40); values ≥ 40 are considered negative. Statistical analysis by Friedman test adjusted with Dunn’s multiple comparisons test: *p < 0.05.

As shown in Fig. 4, according to the time ranges from symptoms onset, we detected MPXV DNA in 7 (70.0%) plasma, serum, and WB out of 10 paired samples collected during week 1 FSO; in 28 (70.0%) plasma, 21 (52.2%) serum, and 15 (37.5%) WB out of 40 paired samples collected during week 2 FSO; and in 3 (27.3%) plasma, 3 (27.3%) serum, and 1 (9.1%) WB out of 11 paired samples collected during week 3 FSO.

For all matrices, the viral load was significantly higher in the first week FSO (plasma: median Ct = 32.0, 95% CI 30.0–> 40; serum: median Ct = 33.1, 95% CI 30.3–> 40; WB: median Ct = 35.2, 95% CI 32.4–> 40) as compared to the following time ranges, when the median Ct values were over 35.

## Discussion

The escalation of clade II MPXV spreading outside the historically endemic countries in Africa during 2022–2023, has raised the level of concern for this emerging disease. Due to its clinical presentation, laboratory confirmation plays a discriminatory role in the diagnosis of MPXV. Various other infections can cause skin affection, and dermal samples should be the first choice for testing the presence of MPXV^16,17^. As in previously published studies^8,12,18,19^, we indeed found the highest viral loads (median Ct: 21.2, 95% CI 20.5–22.3) in skin samples with MPXV DNA positivity rate > 90% in samples collected during week 1 and near to 90% in those collected during week 2. This confirms that skin lesions represent an effective vehicle of transmission in MPXV patients. However, the recent worldwide 2022–2023 outbreak showed that the clinical presentation may be more often atypical and with no-skin-related symptoms^19–22^. In addition, asymptomatic cases have also been reported^23–25^. Non-skin samples can therefore be of great usefulness to support the confirmation of a MPXV diagnosis, investigate possible asymptomatic patients, and monitor viral shedding. In the present study, we extended our previous observation and, in line with previous reports^6–9,26^, showed that viral loads in respiratory tract samples (i.e., saliva and OPS) were found to be much higher than in other clinical materials (i.e., urine, plasma, stool, and semen) during the 4-week follow-up. The viral load decreased markedly in all sample types over time, with median Ct values reaching the negative threshold in urine and plasma during the last time range. In addition, the positivity rates were 95% for both OPS and saliva samples collected during week 1 and still 25% and 31% during week 4, respectively. Semen and stool were detected in 64% and 67% of the samples collected during week 1, with the former showing 30% of samples still positive during week 4. Urine showed the lowest positivity rates during the study period, with MPXV DNA detected in only 30% of samples during week 1. In the present study, we calculated the time to clearance through the Weibull regression model and observed it to be within the third week of illness for all the different clinical samples. The longest median time to MPXV DNA undetectability was observed for OPS at 21 days, followed by saliva at 19 days and stool at 18 days. Semen and urine samples showed similar median time to clearance (14 and 16 days FSO, respectively), but significant differences were observed in viral loads and positivity rates, with urine showing the lowest MPXV DNA levels and number of positive samples at each time point. Notably, aligned with previous findings^6^, long-term shedding was found in semen and saliva, where we detected MPXV DNA after 31 and 71 days, respectively.

Replication-competent virus was cultured from all specimen types, except bloodstream samples (both plasma and serum), likely due to the very low DNA viral loads or presence of immune complexes with IgG. MPXV is known to spread by direct exchange of OPS, but not aerosol. No urine samples were tested for viral culture, as most samples were negative for viral DNA. According to previous data^18,27,28^, the samples resulted in a positive viral culture showed Ct values < 30 (median Ct: 22.4, IQR 21–23) and were collected within the first 10 days of illness (median time: 11 days, IQR 6–11). Although MPXV infection is characterized by two viremic phases, blood samples are not commonly used to confirm MPXV diagnosis; however, investigating the viral kinetics in blood provides important information to better understand the pathogenesis, refine diagnostic algorithms, and improve public health strategies. In fact, MPXV in the blood compartment deserves to be better evaluated to assess whether potential implications for transmission outside the common routes exist, including blood donation and transplantation^29–31^. There are neither documented cases of transfusion-transmitted MPXV nor data on the related blood-born transmission risk^30–33^ and MPXV testing is not included in the screening programs for transfusion-transmissible infections so far. However, MPXV cases have exponentially increased worldwide since May 2022, and the diseases may result in higher severity and morbidity in recipients of blood components and organ transplants^31,34,35^. As for other studies^13,15,18,36^, we detected MPXV DNA at low levels in plasma specimens, with the median Ct value > 30 during week 1 dropping to Ct = 36 during week 2 and > 40 during week 3. These data, together with the lack of isolation of viable viruses in any of the bloodstream samples tested, support the low probability that this sample may be a potential source of infection transmission. The MPXV DNA frequency of detection in bloodstream samples varied among the different studies carried out during the 2022 outbreak^13,15^, ranging from 7 to 88% frequency at diagnosis. Rizzo et al.^36^ showed that 65% of MPXV patients were viremic at diagnosis, having more frequently disseminated skin lesions and a higher percentage of systemic symptoms as compared to non-viremic patients, suggesting that bloodstream dissemination might represent a fundamental step in the disease pathogenesis. In our cohort of MPXV patients, we found that 70% of plasma samples resulted in positive MPXV DNA during weeks 1 and 2, decreasing to 27.3% during week 3; despite the limited number in this last time range, no positive samples collected during week 4 were found to be positive in this study cohort including all MPXV patients with no immunosuppression or AIDS. Prolonged detection of MPXV DNA in plasma (up to 180 days FSO) has been previously reported in immunosuppressed patients who presented a severe and protracted MPXV disease course^37,38^. As in the present study, most of the reports, including data on blood samples collected from MPXV patients, used plasma instead of serum and WB; information on the MPXV detection sensitivity of the three different blood matrices is lacking. Our comparison analysis revealed that plasma showed the best sensitivity for the MPXV molecular assay, reporting the overall highest MPXV DNA detection rate (62% positive samples) and the highest viral loads during the 3-week follow-up as compared to serum and WB. MPXV DNA was detected in 49% of samples of serum, while WB showed the lowest detection rate (38%). One factor responsible for the lower sensitivity found in testing WB may be the higher levels of PCR inhibitors (i.e., haemoglobin and hematin) generally abundant in such sample types, affecting the amplification efficiency of PCR and thus lowering the detection limit^39^. In fact, discordant results were obtained when samples were detected at high Ct values with a median of 36.1. Notably, for some viral pathogens (i.e., Flavivirus)^40,41^, higher rates of nucleic acid detection in WB are observed and are usually due to association to the cellular fractions of blood; in the case of MPXV, the tropism for blood cells should be better defined.

Our results contribute to increasing knowledge and experience with an infection that has been neglected until now and, for the first time, provide information on the testing of the three matrices (plasma, serum, and WB) of bloodstream samples, supporting the use of plasma in cases where there is a need to investigate MPXV blood shedding. The data here presented can be useful for MPXV diagnostics and the control and prevention of its onward transmission, and such analysis should be replicated on clade I MPXV.

## Methods

We tested a total of 723 clinical samples from 89 clade II MPXV-confirmed patients attending the INMI “L. Spallanzani” in Rome, Italy, from May to December 2022. Part of the samples (mainly skin-lesions and OPS) were tested prospectively for diagnostic purposes, while the remaining were tested retrospectively for research purposes. A total of 37 of 84 MPXV patients with available information were people living with HIV (PLWH), with the most recent CD4 count > 200 cells/mm^3^ measured at the MPXV diagnosis (median CD4: 560.5 cells/mm^3^, IQR 412–797.3). Four (4.5%) MPOX patients reported smallpox vaccination during their childhood (median age: 62, IQR 56–62). Of 723 samples, 50 were skin lesions, 109 were OPS, 70 were saliva, 128 were urine, 96 were stool, 147 were semen, and 123 were EDTA plasma. Following the diagnostic confirmation of MPXV infection, the longitudinal study was planned with interval sampling at 7 ± 1, 14 ± 3, 21 ± 3, 28 ± 3, 90 ± 7, 180 ± 30 days from the enrolment day, however, we encountered discontinued participation or availability to provide all specimen types. Specifically, 602 samples (skin, n = 50; OPS, n = 85; saliva, n = 53; urine, n = 93; stool, n = 76; semen, n = 122; EDTA plasma, n = 123) were collected during the 4 weeks follow-up from symptoms onset (FSO) from 37 patients, while 121 (OPS, n = 24; saliva, n = 17; urine, n = 35; stool, n = 20; semen, n = 25) were collected after 30 days up to 4 months FSO. In addition, for 61 bloodstream samples collected from 13 MPXV patients within 3 weeks, we tested paired samples of EDTA-plasma, EDTA-whole blood (WB), and serum. The study was approved by the INMI L. Spallanzani Ethical Committee (approval number: 40z/2022) and informed consent was obtained from all the study patients. The study was performed in accordance with ethical principles for medical research involving human subjects in accordance to relevant national regulations and to the guidelines described by the Declaration of Helsinki.

Swabs from the oropharynx and skin lesions were collected in Universal Transport Medium (UTM-RT®; COPAN Diagnostics, Italy); WB samples were aliquoted using a standard EDTA blood collection tube before the centrifugation step (20 min at 2400×*g*) to collect plasma; saliva samples were collected via passive drooling and spontaneously produced without external stimuli, without the addition of any type of diluent, and at least 30 min after drinking, eating or washing teeth. MPXV DNA was extracted with the QIAsymphony^®^ DSP Virus/Pathogen Midi Kit/the QIAsymphony DSP DNA Mini kit on the QIAsymphony^®^ SP automated platform (QIAGEN, Hilden, Germany) according to manufacturer instructions, with a recovery of 60 ul nucleic acid. The amplification was carried out by means of real-time PCR (RT-PCR), using the protocol published by Li et al.^42^ on RotorGene Q platform. The target was the gene encoding the CrmB secreted TNF-alpha-receptor-like protein of the MPXV genome (G2R gene WA) and the protocol used was specific for clade II MPXV (Green Channel-FAM). RNase P amplification (Red Channel-TXR615) was included as a human sample integrity/extraction control^43^. As a surrogate of the viral DNA levels in the body fluids, we used cycle threshold (Ct) values to study the extent of the MPXV DNA shedding in different biological samples. Samples with Ct values > 40 were considered negative.

Viral culture was performed in the BSL-3 laboratory on Vero E6 cells as described elsewhere^9^. Briefly, samples were diluted in Modified Eagle Medium with l-glutamine (Corning, Glendale, USA) containing a home-made solution of Penicillin G, Streptomycin sulfate, Amphotericin B. The mixtures of sample-antibiotics/antimycotics were kept at room temperature for 30 min and inoculated on Vero E6 cells at the following dilutions: semen 1:10, plasma/serum 1:10, OPS 1:2, saliva 1:10, and stool, which were dissolved in medium and centrifuged, then inoculated in a 1:10 dilution. After 1 h at 37 °C in 5% CO_2_ of incubation, the inoculum was discarded and replaced with MEM containing 2% of heat-inactivated foetal bovine serum (FBS, Corning, Glendale, USA) plus the antibiotics/antimycotics solution. Cytopathic effect (CPE) appearance was observed daily by two independent readers using a light microscope, and in order to evaluate the viral replication, aliquots of cell supernatant were collected at selected time points post-inoculum according to the progress of the infection. In the majority of samples positive for replication-competent virus, CPE was observed between 2 and 6 days.

Continuous variables (i.e., viral loads represented by Ct values and days from symptoms onset) were described using the median and confidence interval 95% (95% CI), while absolute and relative frequencies (i.e., positivity rates) were presented as percentages. Differences in viral loads were described using the Mann–Whitney test to compare MPXV DNA levels in unpaired samples, while the Wilcoxon matched-pairs signed rank test, or the Friedman and Dunn’s multiple comparisons tests were used to compare the viral loads in paired samples. A Weibull regression model was used to calculate the time to viral DNA clearance for each sample type, expressed as the median and 95% CI. Finally, analyses were performed using GraphPad Prism version 9 (GraphPad Software, La Jolla, California, USA) and R version 4.2.2 (R Foundation, Vienna, Austria) with its “survival” package, for Windows statistical software; p < 0.05 was considered statistically significant.

## Data Availability

The data supporting the findings are available only for sections non-infringing personal information, from the corresponding author upon reasonable request.
